# The National Cattle Growers’ Association

**Published:** 1887-01

**Authors:** 


### THE NATIONAL CATTLE GROWERS' ASSOCIATION

Met on October 16th, 1886, in the call room of the Board of Trade, the Hon. D. W. Smith, presiding. This meeting composed of several hundred delegates, who practically represented all the great cattle wealth of the country, the ranch owners of the West, the Shorthorn, Holstein-Friesian, Angus, Jersey, etc., etc., cattle associations, and veterinarians holding official positions or interested in the public weal, was like the preceding meeting, prompt and energetic in its discussions, conclusions and actions.

A year ago the National Cattle Growers' Association had appreciated the necessity of some legislation in regard to contagious diseases, and the more far-seeing members, guided by the energy of the president, Mr. Smith, went to Washington and attempted the passage of a bill, which failed. The recent arrival of the wolf at the very doorstep of the West wakened up every cattle delegate who was present; the lung plague took precedence of every other subject. The resolutions offered by the veterinarians were listened to with attention. Dr. Gadsden, M.R.C.V.S., read two short papers on contagious pleuro-pneumonia. The enthusiasm even reached the proprietors of the Chicago stock yards, and an honest address was presented by Mr. Washburn, the superintendent, who unfortunately, however, supposed that contagious diseases were like a dead whale washed up on a strand, a public nuisance, to be carted away at a certain expense and the carters discharged. He complained of the cost of a bureau (of animal industry) and proposed in its stead a "commission" to be appointed to deal with diseases—presumably a new commission with each outbreak, if not, certainly the permanent "commission" was practically to become a new "bureau." The resolutions presented by the veterinary association were adopted. An error almost occurred in the re-wording of the resolution, in regard to preventive inoculation. The first wording was made to read:

*Resolved*, "That any National legislation for the extirpation "of diseases should prohibit, with appropriate penalties, inocu-"lation with the virus of any contagious diseases incident to "animals,"

But was altered to "Inoculation with the virus of contagious "pleuro-pneumonia."

The following resolution was adopted and a prompt means of furnishing the necessary funds needed for procuring legislation was furnished by a voluntary assessment on the various cattle associations.

*Whereas*, It has been fully demonstrated to this convention by the testimony of professional veterinarians of the highest ability and experience that contagious pleuro-pneumonia exists in an active form among certain cattle in the city of Chicago; and

*Whereas*, From the fatal character of this terrible disease and the difficulty of extirpating it, the existence of it near the greatest cattle market of the United States constitutes a danger to the chief food supply of the country; and

*Whereas*, The further progress of this disease by causing other States to prohibit the introduction of cattle from this

State threatens an entire interruption of inter-State commerce in this vitally important article of trade, and will result in losses of a magnitude that can not be expressed in figures; and

*Whereas,* It is the belief of this convention that a disease so dangerous, and whose consequences would be so disastrous and far-reaching, can not be adequately met and controlled by local authorities acting under state legislation; therefore, be it

*Resolved,* That this convention holds it to be the duty of the national government to undertake the suppression of this disease; that this should be done immediately, in the most thorough and complete manner, and without regard to cost, and that Congress should, at its approaching session, and without a day of unnecessary delay, provide by law appropriate machinery and ample funds for this purpose; and

*Resolved, further,* That a committee of five be appointed by the chair, to be known as the committee on legislation, whose duty it shall be to go to Washington the coming winter and endeavor to obtain the passage of such laws as will accomplish the above end.

*Resolved,* That it is the duty of federal, state and municipal authorities to unite in an effort to extirpate pleuro-pneumonia, wherever it may be found, by destroying diseased cattle, and those having been exposed to disease, so far as possible, purifying all premises in which the disease has existed, and adopting stringent sanitary regulations with regard to the management of cattle in the feeding stations.

This association represents a vast interest at stake, realizes the loss which any further delay in proper legislation entails, and is so thoroughly in earnest, that we may hope for a bill to pass early in the present session of Congress. An important question is what will be the form of the bill? Any satisfactory legislation must provide for a large expenditure of money and settle the question of "States rights."

If this be placed liberally in the hands of the Bureau of Animal Industry, we believe the end will be accomplished. It is sincerely to be hoped that no attempt will be made to form a new commission, to change horses in the middle of the stream, when action must be prompt, when every moment represents one more infecting animal in the country. Our readers are the expert authorities, who have a right, by their education, to guide such legislation as this; and every one should see his representative at Washington, and explain and convince him of the necessity of a liberal bill, not confined to the lung-plague, but looking to the future, controlling all other contagious diseases of our domestic animals. H.

---

Back Numbers Wanted.—We will exchange any number of Vol. VII, (1886), or advance subscription one issue for every copy of the following numbers of this journal, viz.:—Vol. I, Nos. 3 and 4 (July and Oct., 1880); Vol. VI, Nos. 2 and 3 (April and July, 1885).

Please send such copies to the publisher, 1217 Filbert St., Philadelphia, Pa., who will acknowledge their receipt.

---

Semi-Annual Meeting of the United States Veterinary Medical Association. This meeting will be held in Philadelphia, on Tuesday March 15th, 1887. We hope that there will be a full attendance, to furnish, for our next issue, the interesting material which should emanate from the convention of such a society. Many of the older members know Philadelphia only in a social aspect, we trust that both they, and the newer members of the profession will honor their colleagues of Pennsylvania by their presence.

